# Modeling COVID-19 data with a novel neutrosophic Burr-III distribution

**DOI:** 10.1038/s41598-024-61659-2

**Published:** 2024-05-11

**Authors:** Farrukh Jamal, Shakaiba Shafiq, Muhammad Aslam, Sadaf Khan, Zawar Hussain, Qamer Abbas

**Affiliations:** 1 Department of Statistics, The Islamia University of Bahawalpur, Bahawalpur, Pakistan; 2 Department of Statistics, Faculty of Science, King Abdulaziz University, Jeddah, Saudi Arabia

**Keywords:** Burr-III distribution, Nes-BrIII distribution, Neutrosophic analysis, Indetermincy, Cardiology, Health care, Medical research, Engineering, Mathematics and computing

## Abstract

In this study, we have presented a novel probabilistic model called the neutrosophic Burr-III distribution, designed for applications in neutrosophic surface analysis. Neutrosophic analysis allows for the incorporation of vague and imprecise information, reflecting the reality that many real-world problems involve ambiguous data. This ability to handle vagueness can lead to more robust and realistic models especially in situation where classical models fall short. We have also explored the neutrosophic Burr-III distribution in order to deal with the ambiguity and vagueness in the data where the classical Burr-III distribution falls short. This distribution offers valuable insights into various reliability properties, moment expressions, order statistics, and entropy measures, making it a versatile tool for analyzing complex data. To assess the practical relevance of our proposed distribution, we applied it to real-world data sets and compared its performance against the classical Burr-III distribution. The findings revealed that the neutrosophic Burr-III distribution outperformed than the classical Burr-III distribution in capturing the underlying data characteristics, highlighting its potential as a superior modeling toolin various fields.

## Introduction

Historically, in 1942, Burr formulated twelve families of distributions through the Kearl Pearson equation, each offering distinct density functions with diverse applications. Among these, the Burr-III distribution has gained widespread acceptance and recognition. However, the Burr-III distribution has often been overlooked in favor of other Burr family of distributions. Examples of its applications include forestry studies by Gove et al.^1^, life testing investigations by Wing^2,3^, operational risk assessments by Chernobai et al.^4^, analyses of option market price distributions by Sherrick et al.^5^, metrological studies by Mielk^6^, crop yield modeling by Tejeda and Goodwin^7^, and reliability assessments by Abdel-Ghaly et al.^8^. The Burr-III distribution often referred to as the Dagum distribution in income and earning studies (as observed in^9^) is significant component in the field of science. In the realm of real-world applications, it is known as the inverse Burr-XIII distribution (as highlighted in^10^). Benjamin et al.^11^ had leveraged the Dagum distribution to model maximum daily levels of troposphere ozone, demonstrating the versatility and relevance of the Burr-III distribution in various scientific domains. The cumulative distribution function (CDF) of the classical Burr-III distribution is given as:

1 $$G(x;\theta ,\lambda ) = \left( {1 + x^{ - \theta } } \right)^{ - \lambda } ,\,\,\,\,\,\,\lambda ,\theta > 0,\,\,x > 0.$$

Despite the widespread use of the Burr-III distribution, existing research work has encountered certain limitations with the classical Burr-III distribution as:

1. One primary challenge is its inability to effectively capture the complexities of real-world datasets characterized by uncertainty and ambiguity. Many complex problems inherently involve vague and imprecise information, which classical Burr-III model often struggle to address adequately.
2. The classical Burr-III model assumed that data is crisp and well-defined, which may not always hold true in practice. This discrepancy between the assumptions of classical Burr-III model and the nature of real-world data introduces a significant source of error and uncertainty in statistical analyses.

To address these limitations, there is a growing need for alternative approaches that can accommodate uncertainty and indeterminacy in data analysis. One promising paradigm shift in this regard is the field of neutrosophic analysis, which introduces a novel approach for handling data uncertainty.

The concept of “Neutrosophic Statistics” introduces a novel approach for handling data uncertainty. It encompasses both uncertain data and the methodologies employed to evaluate such data. The distinguishing feature of neutrosophic statistics lies in its ability to accommodate uncertainty and indeterminacy, a departure from classical statistics where all data is crisp and well-defined. Neutrosophic statistics come into play when data contains indeterminacy, offering valuable tools for analyzing such uncertain information. Numerous neutrosophic probability distributions have been developed in the literature, for instance, neutrosophic Weibull distribution by Al-hasan and Smarandache^12^, neutrosophic uniform, neutrosophic exponential and neutrosophic Poisson by Al-habib et al.^13^, neutrosophic normal and neutrosophic binomial distributions by Pareto and Smarandache^14^, neutrosophic Rayleigh distribution by Aslam^15^ and neutrosophic Beta distribution by Sherwani et al.^16^.

In this study, we aim to introduce a ground breaking probabilistic model known as the Netrosophic Burr-III (NeS-BrIII) distribution, specifically designed to tackle the intricacies of neutrosophic surface analysis. Our exploration into the NeS-BrIII distribution delves deep into its capabilities, unveiling its potential to address the challenges posed by ambiguity and vagueness in the data that the classical Burr-III distribution cannot meet these challenges. This unique distribution not only offers insights into reliability properties, moment expressions, order statistics, and entropy measures but also serves as a versatile tool for unraveling the complexities of real-world datasets. To gauge the practical significance of our proposed NeS-BrIII distribution, we applied it to real-world datasets and subject it to rigorous performance evaluation. By comparing its results against those of the classical Burr-III distribution, we aim to showcase the NeS-BrIII distribution’s prowess in capturing the nuanced characteristics of data, thereby highlighting its potential as a superior modeling tool across a spectrum of fields and applications.

The structure of the paper is formatted as: Section "The model with properties" laid the foundation for the current study by presenting the development of the NeS-BrIII distribution. We have derived the extensive properties of the NeS-BrIII distribution. We delved into its characteristics and conduct simulations to further elucidate its behavior. This section serves as a comprehensive examination of the distribution’s features. In Section "Applications of the NeS-BrIII distribution", we applied the NeS-BrIII distribution to real-world data sets, demonstrating its practical utility. Through these applications, we showcase how the distribution can be effectively employed to analyze and model real data, offering insights into its performance and versatility. The final section, Section "Conclusion", serves as the conclusion of our study by summarizing the key findings, contributions and implications of our research.

## The model with properties

In this section, we delve into the development of the neutrosophic model based on the classical Burr-III distribution. This section provides a framework for the development of the NeS-BrIII distribution as:

1. The first step in developing the NeS-BrIII distribution involves extending the classical Burr-III distribution to accommodate neutrosophic concepts. This extension involves incorporating neutrosophic parameters and defining neutrosophic versions of PDF and CDF as: $F_{Neu} (x;\lambda_{Neu} ,\alpha_{Neu} ) = F_{L} [(X_{L} + X_{L} I_{Neu} );\lambda_{L} ,\alpha_{L} )$ and $f_{Neu} (x;\lambda_{Neu} ,\alpha_{Neu} ) = f_{L} [(X_{L} + X_{L} I_{Neu} );\lambda_{L} ,\alpha_{L} )$.
2. After model development, the second step involves investigating the properties and characteristics of the NeS-BrIII distribution, including moments, reliability properties, order statistics, and entropy measures. These analyses provide insights into the behavior and performance of the distribution in various contexts.
3. Next, the maximum likelihood estimation method along with simulation is being tailored to the NeS-BrIII distribution. This method leverage neutrospohic statistics to estimate the parameters of the distribution from the observed data

### The model development

If the random variable X follow the Burr-III distribution having CDF defined in Eq. (1) and considering that $Neu$ is a neutrosophic statistical number and that $I_{Neu} \in \left( {I_{L} ,I_{N} } \right)$ is an interval of indeterminacy. Such that $X_{Neu} \in \left( {n_{L} ,n_{N} } \right)$ if the neutrosophic variable $X_{Neu} \in \left( {X_{L} ,X_{N} } \right)$ generates neutrosophic observations. Hence the neutrosophic form can be expressed as: $X_{Neu} = X_{L} + X_{L} I_{Neu}$. Where, in $I_{Neu} \in \left( {I_{L} ,I_{N} } \right)$, $I_{L}$ and $I_{N}$ represents the classical and neutrosophic statistics, respectively. Similarly, in $X_{Neu} \in \left( {n_{L} ,n_{N} } \right)$ and $X_{Neu} \in \left( {X_{L} ,X_{N} } \right)$; $n_{L} ,X_{L}$ represent the classical statistic and $n_{N} ,X_{N}$ represent the neutrosophic statistics.

Thus the CDF of the NeS-BrIII distribution can be defined as:

2 $$F(x;\lambda_{Neu} ,\theta_{Neu} ) = \left\{ {1 + \left[ {(1 + I_{Neu} )x} \right]^{{ - \theta_{Neu} }} } \right\}^{{ - \lambda_{Neu} }} ,\,\,\,\lambda_{Neu} ,\theta_{Neu} > 0,x > 0.$$

**Figure 1:**
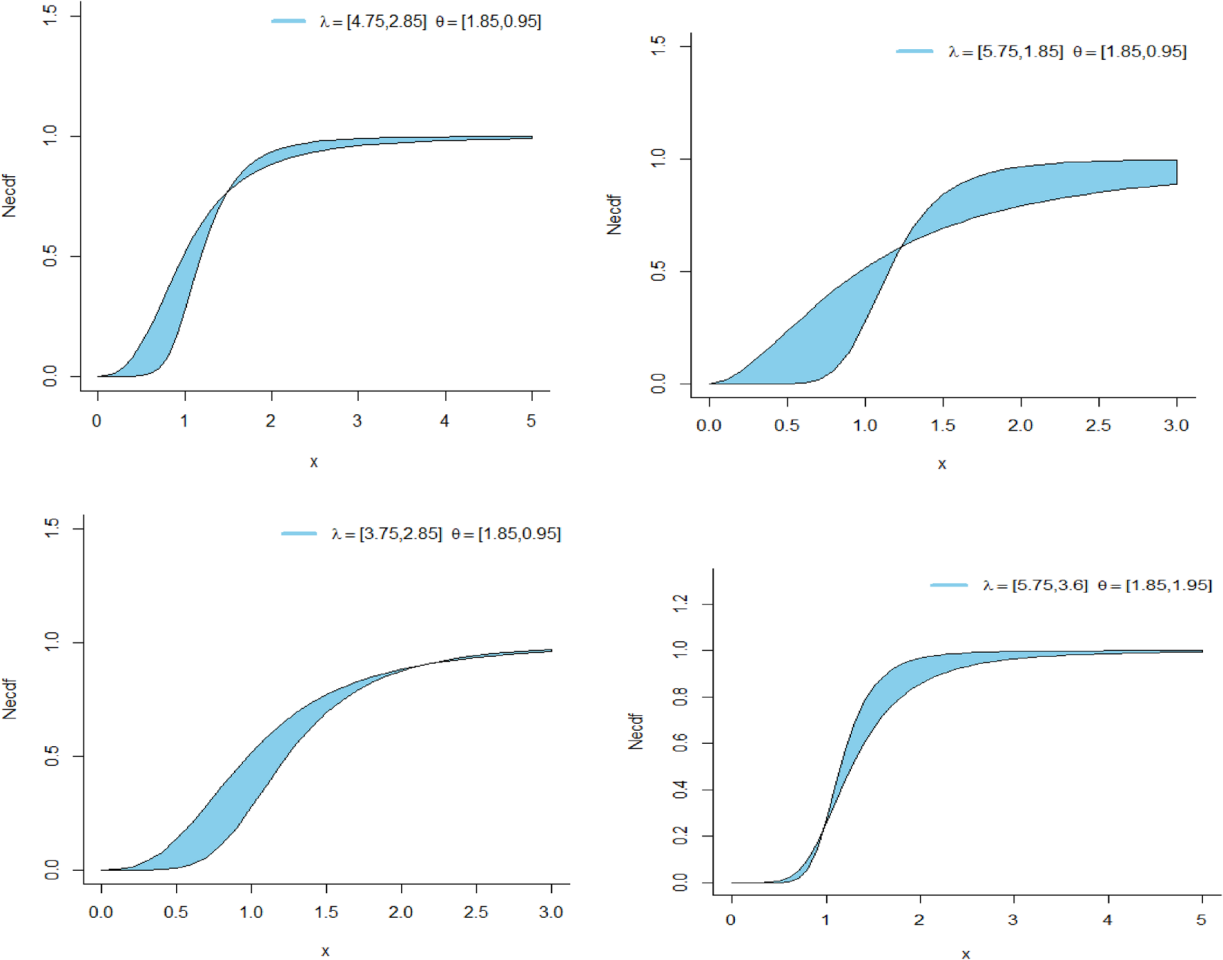
CDF plots of NeS-BrIII distribution.

Here $\lambda_{Neu} ,\theta_{Neu}$ are the shape parameters. The CDF graphs of the NeS-BrIII distribution given in Fig. 1 are representing an increasing function of the CDF of the NeS-BrIII distribution. The probability density function (PDF) of the NeS-BrIII distribution derived from Eq. (2) is expressed as:

3 $$f(x;\lambda_{Neu} ,\theta_{Neu} ) = \left( {1 + I_{Neu} } \right)^{{ - \theta_{Neu} }} \lambda_{Neu} \theta_{Neu} x^{{ - \theta_{Neu} - 1}} \left\{ {1 + \left[ {(1 + I_{Neu} )x} \right]^{{ - \theta_{Neu} }} } \right\}^{{ - \lambda_{Neu} - 1}} ,$$

**Figure 2:**
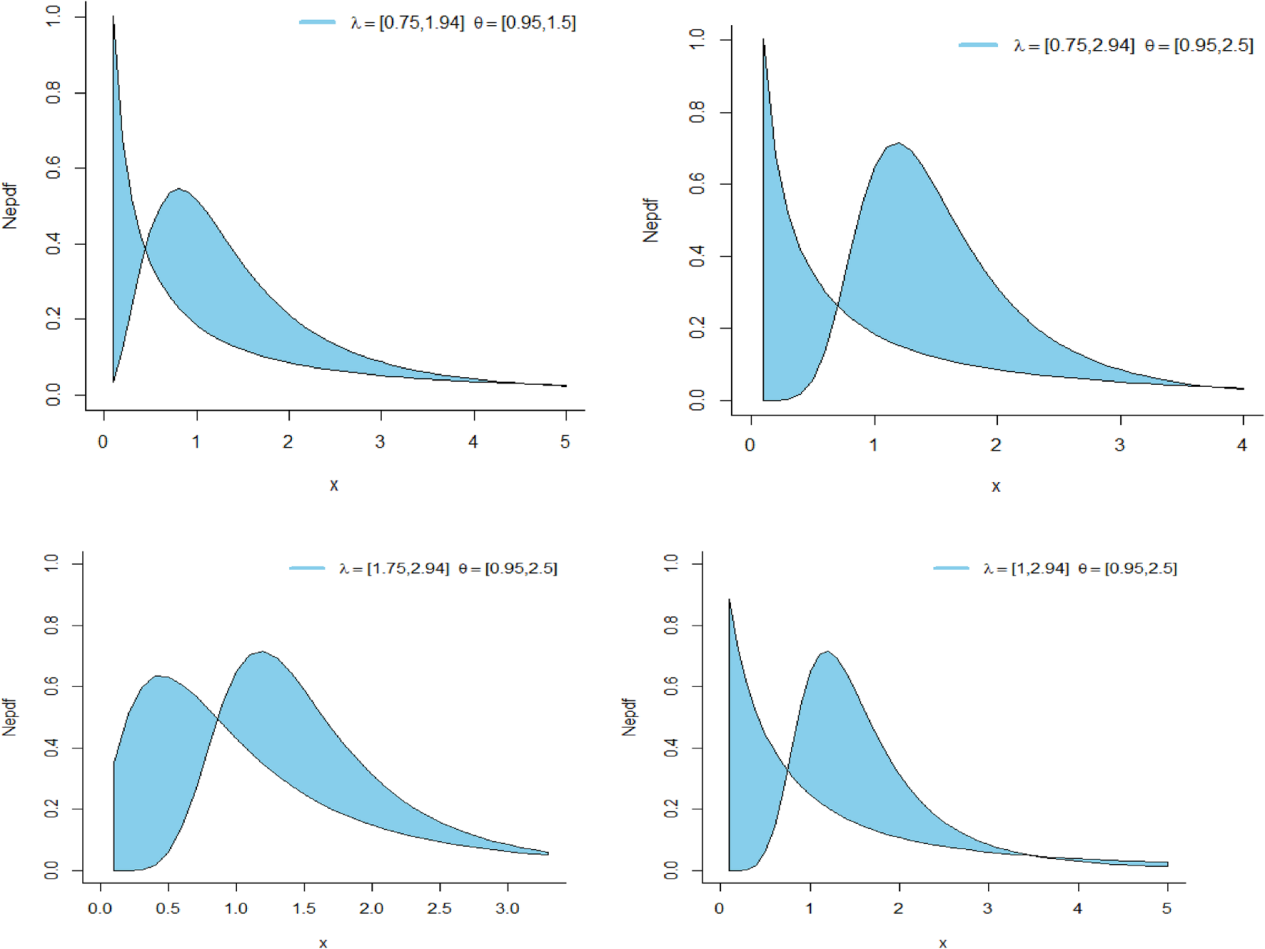
PDF plots of NeS-BrIII distribution.

The PDF plots of the NeS-BrIII distribution are being presented in Fig. 2. Notably, the PDF plots of the NeS-BrIII model prominently display aunimodel behavior, indicating a central tendency within the variable being modeled. This unimodel nature of the model suggests that the distribution predominantly exhibit a single peak, signifying a concentration of values around a specific point.

The survival function (SF) and hazard rate function (HRF) of the NeS-BrIII model are respectively expressed given below

4 $$S(x;\lambda_{Ne} ,\theta_{Neu} ) = 1 - \left\{ {1 + \left[ {(1 + I_{Neu} )x} \right]^{{ - \theta_{Neu} }} } \right\}^{{ - \lambda_{Neu} }} ,$$

and

5 $$h(x;\lambda_{Ne} ,\theta_{Neu} ) = \frac{{\left( {1 + I_{Neu} } \right)^{{ - \theta_{Neu} }} \lambda_{Neu} \theta_{Neu} x^{{ - \theta_{Neu} - 1}} \left\{ {1 + \left[ {(1 + I_{Neu} )x} \right]^{{ - \theta_{Neu} }} } \right\}^{{ - \lambda_{Neu} - 1}} }}{{1 - \left\{ {1 + \left[ {(1 + I_{Neu} )x} \right]^{{ - \theta_{Neu} }} } \right\}^{{ - \lambda_{Neu} }} }}.$$

**Figure 3:**
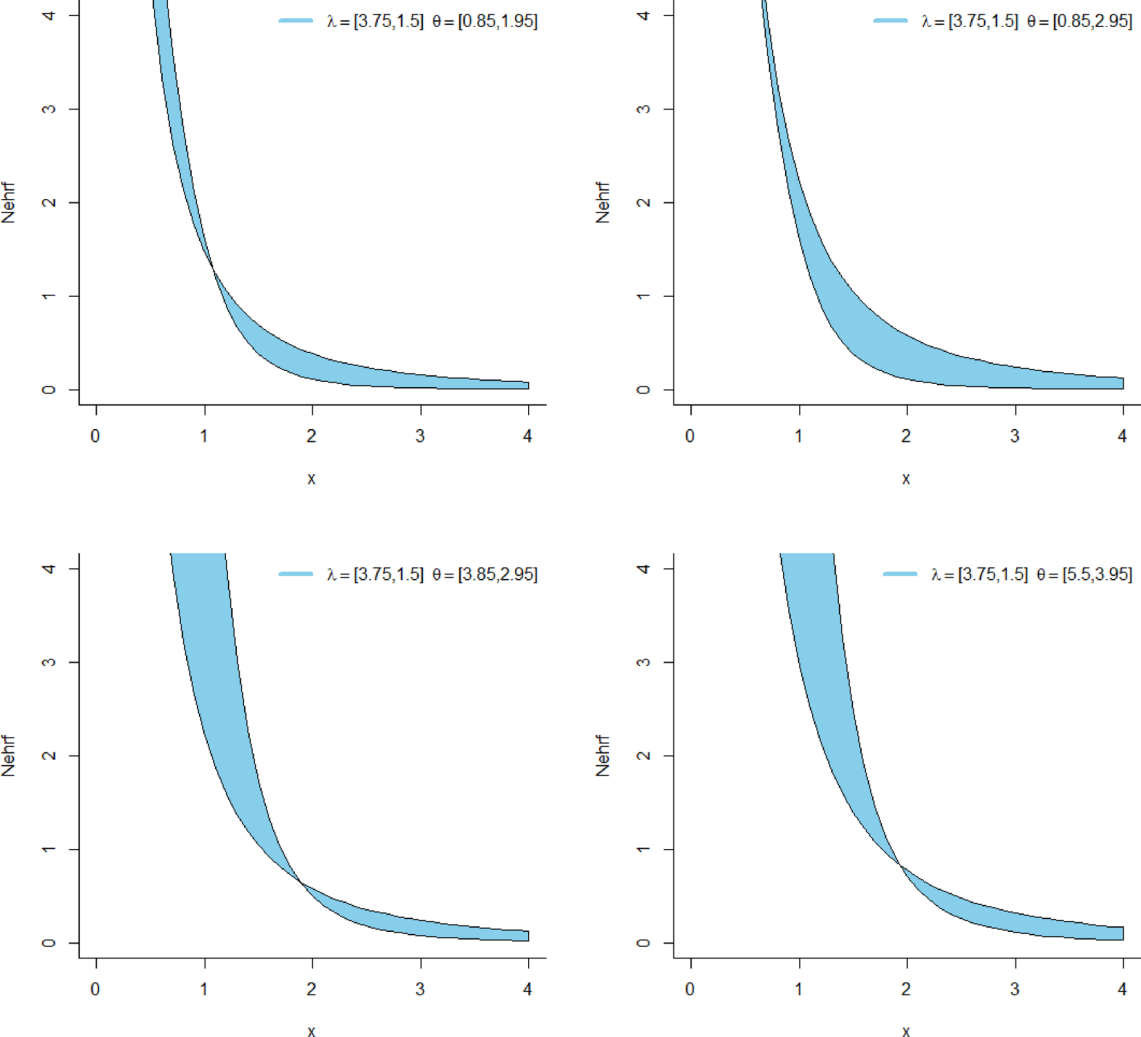
HRF plots of the NeS-BrIII distribution.

Figure 3 represents the HRF plots of the NeS-BrIII distribution. The HRF plots of the NeS-BrIII model vividly portray two distinct shapes: a decreasing pattern and a bathtub pattern. These two shapes offer valuable insights into the distribution’s behavior, highlighting both the initial decline in HRF by a resurgence as a unique characteristic of the NeS-BrIII distribution.

### Properties and simulation

The NeS-BrIII distribution offers a wealth of properties and computations that significantly enhance the understanding of its practical implementation within distribution theory. Key properties such as moments, percentiles, random number generation, as well as maximum likelihood estimation (MLE) and dependability measures have been rigorously determined for the NeS-BrIII distribution. These calculated properties and measures collectively contribute to a comprehensive characterization of the NeS-BrIII distribution, enhancing its utility in various theoretical and practical contexts within distribution theory.

#### Moments of the NeS-BrIII distribution

Moments serve as essential statistical measures that bridge theory and observations. Moments are calculated values that describe key properties of a distribution, specifically the expected values of different powers of the random variable. In practice, moments reveal crucial insights into the distribution’s behavior and characteristics. To apply moments to real-world data, we adjust them to match sample moments. This adjustment process involves equating estimated moments from observed data to their corresponding population moments. The number of equations generated in this process equals the number of parameters being estimated. One prominent application of moments is Pearson’s correlation coefficient, which establishes a connection between numerical moments of a distribution and population moments. This coefficient is widely used for analyzing relationships within data is fundamental in statistical analysis.

The $r$ ^th^ moments of NeS-BrIII distribution are

6 $$\mathop \mu \nolimits_{r}^{\prime } = \left( {1 + I_{Neu} } \right)^{r} \int\limits_{0}^{\infty } {x_{L}^{r} } f(x_{L} )dx_{L} = \lambda_{Neu} \left( {1 + I_{Neu} } \right)^{r} {\text{B}}({\text{A}}_{r} ,{\text{B}}_{r} ),\,\,\,\,\,r = 1,2, \ldots$$

where B(A$r$,B$r$) is a beta function, A$r$ = 1 − $\frac{r}{{\theta_{Neu} }}$, B$r$ = $\lambda_{Neu}$ + $\frac{r}{{\theta_{Neu} }}$ and $r$ < $\theta_{Ne}$.

The negative moments of NeS-BrIIIare

7 $$\mathop \mu \nolimits_{ - r}^{\prime } = \left( {1 + I_{Neu} } \right)^{r} \lambda_{Neu} B\left( {\frac{r}{{\theta_{Neu} }} + 1,\lambda_{Neu} - \frac{r}{{\theta_{Neu} }}} \right),\,\,r = {1},{2}, \ldots$$

For $$\lambda_{Neu} ,\theta_{Neu} > r$$.

The mean and variance of NeS-BrIII distribution are given as:

8 $$E(X) = \left( {1 + I_{Neu} } \right)\lambda_{Neu} B\left( {\frac{1}{{\theta_{Neu} }} + 1,\lambda_{Neu} - \frac{1}{{\theta_{Neu} }}} \right),$$

And

9 $$Var(X) = \left( {1 + I_{Neu} } \right)^{2} \lambda_{Neu} \left[ {B\left( {\frac{2}{{\theta_{Neu} }} + 1,\lambda_{Neu} - \frac{2}{{\theta_{Neu} }}} \right) - \lambda_{Neu} B^{2} \left( {\frac{1}{{\theta_{Neu} }} + 1,\lambda_{Neu} - \frac{1}{{\theta_{Neu} }}} \right)} \right].$$

Skewness (C_1_) and kurtosis (C_2_) can be computed from the following moment ratios:

10 $${\text{C}}_{{1}} = \frac{{E(X^{3} ) - 3E(X)E(X^{2} ) + 2E^{3} (X)}}{{Var\frac{3}{2}(X)}}$$

11 $${\text{C}}_{{2}} = \frac{{E(X^{4} ) - 4E(X)E(X^{3} ) + 6E(X^{2} )E^{2} (X) - 3E^{4} (X)}}{{Var^{2} (X)}}$$

Table 1 provides a comprehensive overview of computed statistical first four moments, variance (V), skewness (C_1_) and kurtosis (C_2_) for the some arbitrary parametric values of NeS-BrIII distribution using R-Programming language.

**Table 1:** The numerical values of the first four moments ($\mu_{r}{\prime}$, r = 1,2,3,4), V, C_1_and C_2_ of the NeS-BrIII model for some parameter values.

$\lambda_{Neu} ,\theta_{Neu}$	$\mu_{1}{\prime}$	$\mu_{2}{\prime}$	$\mu_{3}{\prime}$	$\mu_{4}{\prime}$	V	C_1_	C_2_
[0.1,0.2],[0.2,0.3]	[0.010,0.028]	[0.004,0.013]	[0.003,0.009]	[0.002,0.006]	[0.004, 0.012]	[9.283,5.351]	[112.20,47.98]
[0.3,0.4],[0.4,0.5]	[0.053,0.081]	[0.025,0.040]	[0.016,0.026]	[0.012,0.019]	[0.022,0.033]	[3.761,2.889]	[32.74,27.530]
[0.5,0.6],[0.6,0.7]	[0.109,0.136]	[0.055,0.070]	[0.036,0.047]	[0.027,0.035]	[0.043,0.052]	[2.335,1.954]	[25.679, 25.108]
[0.7,0.8],[0.8,0.9]	[0.160,0.179]	[0.086,0.100]	[0.058,0.068]	[0.043, 0.051]	[0.060, 0.067]	[1.678,1.475]	[24.911,24.641]
[0.9,1],[1,1.1]	[0.194,0.205]	[0.112,0.123]	[0.077,0.086]	[0.059,0.066]	[0.074, 0.080]	[1.325,1.219]	[24.113,23.305]
[1.1,1.2],[1.2,1.3]	[0.212,0.216]	[0.131,0.138]	[0.094,0.100]	[0.072,0.078]	[0.086,0.091]	[1.148,1.108]	[22.286, 21.157]
[1.3,1.4],[1.4,1.5]	[0.217, 0.216]	[0.143, 0.146]	[0.105,0.109]	[0.083,0.087]	[0.096,0.099]	[1.092,1.098]	[20.009,18.910]
[1.5,1.6],[1.6,1.7]	[0.212, 0.208]	[0.148,0.148]	[0.112,0.114]	[0.090,0.092]	[0.102,0.105]	[1.121,1.157]	[17.903,17.010]
[1.7,1.8],[1.8,1.9]	[0.202,0.195]	[0.147,0.145]	[0.115,0.115]	[0.093,0.094]	[0.106,0.107]	[1.205,1.263]	[16.24,15.592]

#### Quantile function and random numbers generation

In general the quantile function (QF) is a mathematical expression used to determine specific quantiles of the distribution. In the realm of probability distribution, a distribution function typically adheres to two important characteristics: it is a non-decreasing function, and its QF is left-continuous. The QF maps values from the range [0, 1], representing the probabilities. The QF of the NeS-BrIII distribution is

12 $$x_{q} = (1 + I_{Neu} )^{ - 1} \left[ {q^{{\frac{1}{{\lambda_{Neu} }}}} - 1} \right]^{{ - \frac{1}{{\theta_{Neu} }}}} .$$

When we set the quantile value to 0.5 in Eq. (13), we obtain the median of the NeS-BrIII distribution. The median is a crucial measure that divides the distribution into two equal halves, making it an essential indicator of central tendency in this particular distribution.

#### Reliability function of the NeS-BrIII distribution

Reliability is often defined as a device's capacity to work under a specific set of circumstances until it fails. Strength that can withstand stress under constant operating conditions is included into several of the devices' designs. The link between stress and strength is complex. Engineering, medicine, sociology, and other fields of study have all examined it. If $P(X<Y), P(X<Z),$ and other probability are estimated. This, nevertheless, the "stress-strength" or "reliability" models are a collection of probabilistic models.

If $X_{1}$ represents a stochastic element's strength and $X_{2}$ represents its stress, the stress strength model characterizes the unpredictable element's life. When the component is subjected to stress that exceeds its strength, it will fail, but it will still work properly. Whenever $X_{2}$ < $X_{1}$, hence

13 $$R = P(X_{2} < X_{1} ) = \int\limits_{0}^{\infty } {f_{1} (x)F_{2} (x)dx} ,$$

where R is a measure of reliability.

Let $$X_{1}$$ and $$X_{2}$$ be the independent NeS-BrIII distributions’ variables as:$$X_{1}$$ follows the NeS-BrIII($$\lambda_{Neu1}$$$$,\theta_{Neu}$$) and $$X_{2}$$ follows the NeS-BrIII($$\lambda_{Neu2}$$$$,\theta_{Neu}$$). Then fromEquations (3) and (4), we have

14 $$R = (1 + I_{Neu} )\lambda_{Neu1} \theta_{Neu} \int\limits_{0}^{\infty } {\left[ {(1 + I_{Neu} )x} \right]^{{ - (\theta_{Neu} + 1)}} \left\{ {1 + \left[ {(1 + I_{Neu} )x} \right]^{{ - \theta_{Neu} }} } \right\}}^{{ - (\lambda_{Neu1} + \lambda_{Neu2} ) - 1}} d(x) = \frac{{(1 + I_{Neu} )\lambda_{Neu1} }}{{\lambda_{Neu1} + \lambda_{Neu2} }}.$$

#### Ordrer statistics of the NeS-BrIII distribution

Order statistics are essential in data analysis and their features. Statistical implementations have been extensively researched in the literature.The oldest model for ordered random variables is probably order statistics. When observations in a sample are ordered in increasing order of size, order statistics emerge naturally in life.Order statistics is also useful for studying distribution of maximum, minimum and median etc. Based on random variables, let $X_{1} ,X_{2} ,...,X_{n} ,$ follows the NeS-BrIII density function with absolutely continuous distribution function, the ith order statistics of NeS-BrIII is defined as:

15 $$f_{{x_{(j)} }} (x) = \frac{{(1 + I_{Neu} )^{j + k} \lambda_{Neu} \theta_{Neu} n!}}{(j - 1)!(n - j)!}\sum\limits_{k = 0}^{n - j} {( - 1)^{k} } \left( \mathop {_ {k}^{n - j}}  \right)x^{{ - \theta_{Neu} - 1}} (1 + x^{{ - \theta_{Neu} }} )^{{ - \lambda_{Neu} (j + k) - 1}} .$$

Ith order moments of NeS-BrIII is

16 $$\begin{gathered} E(\mathop x\nolimits_{(j)}^{r} ) = \frac{{(1 + I_{Neu} )^{j + k} \lambda_{Neu} \theta_{Neu} n!}}{(j - 1)!(n - j)!}\sum\limits_{k = 0}^{n - j} {( - 1)^{k} } \left( \mathop {_ {k}^{n - j}}  \right)\int\limits_{0}^{\infty } {x^{{r - \theta_{Neu} - 1}} } (1 + x^{{ - \theta_{Neu} }} )^{{ - \lambda_{Neu} (j + k) - 1}} dx \hfill \\ = E(\mathop x\nolimits_{(j)}^{r} ) = \frac{{(1 + I_{Neu} )^{j + k} \lambda_{Neu} n!}}{(j - 1)!(n - j)!}\sum\limits_{k = 0}^{n - j} {( - 1)^{k} } \left( \mathop {_ {k}^{n - j}}  \right)B\left( {1 - \frac{r}{{\theta_{Neu} }},\frac{r}{{\theta_{Neu} }} + \lambda_{Neu} (j + k)} \right). \hfill \\ \end{gathered}$$

#### *Renyi’s* entropy

An entropy is a numerical measure of a system's uncertainties. The higher the entropy, the more unpredictable the data. The entropy of Renyi is defined as:

17 $$I_{R} (\varepsilon ) = \frac{1}{1 - \varepsilon }\log \left[ {I(\varepsilon )} \right],$$

From Eq. (3), we have

18 $$I(\varepsilon ) = (1 + I_{Neu} )^{\varepsilon } (\lambda_{Neu} \theta_{Neu} )^{\varepsilon } \int\limits_{0}^{\infty } {x^{{ - \varepsilon (\theta_{Neu} + 1)}} } \left[ {1 + x^{{ - \theta_{Neu} }} } \right]^{{ - \varepsilon \left( {\lambda_{Neu} + 1} \right)}} dx$$

19 $$= \mathop {(1 + I_{Neu} )^{\varepsilon } \lambda }\nolimits_{{_{Neu} }}^{\varepsilon } \mathop \theta \nolimits_{{_{Neu} }}^{\varepsilon - 1} B\left( {\varepsilon \left( {1 + \frac{1}{{\theta_{Neu} }}} \right) + 1,\varepsilon \left( {\lambda_{Neu} + \frac{1}{{\theta_{Neu} }}} \right) - 1} \right).$$

#### Maximum likelihood estimation

MLE is a fundamental statistical method used to estimate the parameters of a statistical model by maximizing the likelihood function. MLE provides efficient and asymptotically unbiased estimates, and its widespread use in various fields underscores its importance as a robust and consistent method for parameter estimation. The log likelihood function of the Nes-BrIII model is

$$L(\theta_{Neu} ,\lambda_{Neu} ) = n\log \theta + n\log \lambda_{Neu} - n\theta_{Neu} \log (1 + I_{Neu} ) + ( - \theta_{Neu} - 1)\sum\limits_{i = 1}^{n} {\log x_{i} + ( - \lambda_{Neu} - 1)\sum\limits_{i = 1}^{n} {\log \left\{ {\left[ {(1 + I_{Neu} )x_{i} } \right]^{{ - \theta_{Neu} }} } \right\}} }$$

Taking derivative of $L(\theta_{Neu} ,\lambda_{Neu} )$ with respect to $\theta_{Neu} ,\lambda_{Neu}$, and equating the resulting expressions to zero will yield the MLE(s) of the NeS-BrIII model as:

$$L_{\theta } = \frac{n}{\theta } - n\log (1 + I_{Neu} ) - \sum\limits_{i = 1}^{n} {x_{i} + (\lambda + 1)} \sum\limits_{i = 1}^{n} {\log \left[ {(1 + I_{Neu} )x_{i} } \right]} = 0$$

$$L_{\lambda } = \frac{n}{\lambda } - \sum\limits_{i = 1}^{n} {\log \left[ {((1 + I_{Neu} )x_{i} )^{ - \theta } } \right]} = 0$$

As the above expressions are non-linear equations so the numerical integration is applied using R programming language to compute the MLE(s) of the NeS-BrIII model.

#### Simulation study

In this section, we carried out a simulation study to check the behavior of proposed estimators for the NeS-BrIII model.

1. We have generated 5000 samples of sizes, *n* = 30, 50, 100, 200 and 300 from the NeS-BrIII distribution with different combinations of parameters.

2. The average estimates (AEs), biases and mean square error (MSEs) are computed to check the performance of the best estimator as:

$$AEs = \sum\limits_{i = 1}^{N} {\hat{v}_{i} } /5000,Bias = \sum\limits_{i = 1}^{N} {\left( {\frac{{\hat{v}_{i} }}{5000}} \right)} - v_{i} ,MSE = \sum\limits_{i = 1}^{N} {\left( {\hat{v}_{i} - v_{i} } \right)}^{2} /5000$$

3. The results of the simulation study are listed in Tables 2, 3 and 4. The results given in the Tables 2, 3 and 4 showed that MLEs are consistent estimators. The biases and MSEs decreased by increased in the sample size. Moreover, the results of the NeS-BrIII model are more precise and error free as presented in the form of interval.

**Table 2:** Parameter estimates for $\lambda_{Neu}$ = [0.5, 1] and $\theta_{Neu}$ = [3, 2].

n	AEs		Biases		MSEs	
	$\lambda_{Neu}$	$\theta_{Neu}$	$\lambda_{Neu}$	$\theta_{Neu}$	$\lambda_{Neu}$	$\theta_{Neu}$
30	[0.5803, 2.1940]	[6.6985, 4.1667]	[0.0803, 0.6940]	[3.6985,0.1667]	[0.8829, 156.3868]	[18.978, 159.7938]
50	[0.6115, 2.0949]	[6.4642, 3.7285]	[0.1115, 0.5949]	[3.4642,0.2715]	[0.4398, 164.7720]	[14.492, 161.7065]
100	[0.6228, 2.3596]	[6.3480, 5.1059]	[0.1228, 0.8596]	[3.3480,1.1059]	[0.2387, 172.6290]	[12.505, 179.7079]
200	[0.6528, 2.5516]	[6.3511, 2.5953]	[0.1528, 1.0516]	[3.3511,-1.4047]	[0.0247, 161.0996]	[11.462, 155.3355]
300	[0.6501, 1.7067]	[6.3340, 4.8442]	[0.1501, 0.2067]	[3.3340,0.8442]	[0.0234, 170.3878]	[11.2413, 172.796]

**Table 3:** Parameter estimates for $\lambda_{Neu}$ = [2, 3] and $\theta_{Neu}$ = [5, 6].

n	AEs		Biases		MSEs	
	$\lambda_{Neu}$	$\theta_{Neu}$	$\lambda_{Neu}$	$\theta_{Neu}$	$\lambda_{Neu}$	$\theta_{Neu}$
30	[2.5467, 3.205]	[5.279, 5.1355]	[0.5467, 0.205]	[0.2791, − 0.8645]	[165.125, 164.698]	[186.712, 146.370]
50	[1.9374, 2.1365]	[5.2322, 6.6268]	[− 0.0626, − 0.8635]	[0.2322, 0.6268]	[172.641, 168.386]	[161.902, 174.045]
100	[1.9364, 1.7966]	[5.0903, 5.8185]	[− 0.0636, − 1.2034]	[0.0903, 0.1815]	[174.680, 170.570]	[155.784, 169.080]
200	[1.4211, 3.2801]	[5.7962, 5.6657]	[− 0.5789, 0.2801]	[0.7962, 0.3343]	[147.449, 162.112]	[154.387, 170.439]
300	[0.9290, 1.8930]	[4.2522, 5.4604]	[− 1.0710, − 1.1070]	[− 0.7478, 0.5396]	[184.149, 168.487]	[181.309, 174.949]

**Table 4:** Parameter estimates for $\lambda_{Neu}$ = [4, 4.5] and $\theta_{Neu}$ = [7, 7.5].

n	AEs		Biases		MSEs	
	$\lambda_{Neu}$	$\theta_{Neu}$	$\lambda_{Neu}$	$\theta_{Neu}$	$\lambda_{Neu}$	$\theta_{Neu}$
30	[4.5841, 4.7289]	[6.7477, 7.0813]	[0.5841, 0.2289]	[0.2523, 0.4187]	[172.366, 182.541]	[158.872, 176.007]
50	[4.0612, 4.9979]	[6.4350, 7.8684]	[0.0612, 0.3684]	[− 0.5650, 0.3684]	[181.070, 158.172]	[176.790, 160.662]
100	[5.1519, 2.9119]	[8.2333, 7.2606]	[1.1519, − 1.5881]	[1.2333, − 0.2394]	[175.365, 175.512]	[195.365, 165.819]
200	[4.2100, 4.9594]	[6.9854, 7.4938]	[0.2100, 0.4594]	[0.0146, − 0.0062]	[172.806, 169.929]	[188.124, 163.348]
300	[3.9717, 4.2411]	[6.5841, 6.7488]	[− 0.0283, − 0.2589]	[− 0.4159,− 0.7512]	[191.327, 177.967]	[157.121, 177.275]

## Applications of the NeS-BrIII distribution

An application on two actual data setsis being presented in this section.We have converted the classical data into neutrosophic form to deal with the imprecision, uncertainty or ambiguity in the classical data. Table 5 represents the crisp values for data set 1 and 2, respectively, by converting the classical data into neutrosophic form by letting I_Neu_ = [0, 0.05] in X_Neu_ = [X_L_, X_L_(1 + I_N_)]. The lower values represent the classical statistics and the upper values represent the neutrosophic statistics in the interval form of the data. We considered several goodness of fit criterion that allowed us to compare the fits of the NeS-BrIII by considering I_Neu_ = 0.05and classical Burr-III by considering I_Neu_ = 0 models numerically. Among these information criteria (ICs), Akaike information criterion (AIC), corrected Akaike information criterion (CAIC), Bayesian information criterion (BIC) and Hannan-Quinn information criterion (HQIC) were used to choose the best model. The lower the values of AIC, CAIC, BIC and HQIC, shows the better fit of the model. The fits of the NeS-BrIII model were compared with the well-known classical models namely Burr-III, Burr-XII, Weibull (W) and Nadarajah and Haghighi (NH) models.

**Table 5:** The data values for the data 1 and data 2.

Data 1	Data 2
(14.918, 15.66390), (10.056, 11.18880), (12.274, 12.88770), (10.289, 10.80345), (10.832, 11.37360), (7.099, 7.45395), (5.928, 6.22440), (13.211, 13.87155), (7.968, 8.36640), (7.584, 7.96320), (5.555, 5.83275), (6.027, 6.32835), (4.097, 4.30185), (3.611, 3.79155), (4.960, 5.20800), (7.498, 7.87290), (6.940, 7.28700), (5.307, 5.57235), (5.048, 5.30040), (2.857, 2.99985), (2.254, 2.36670), (5.431, 5.70255), (4.462, 4.68510), (3.883, 4.07715), (3.461, 3.63405), (3.647, 3.82935), (1.974, 2.07270), (1.273, 1.33665), (1.416, 1.48680), (4.235, 4.44675)	(1.1, 1.155), ( 1.4, 1.470), (1.3, 1.365), (1.7, 1.785), (1.9, 1.995), (1.8, 1.890), (1.6, 1.680), (2.2, 2.310), (1.7, 1.785), (2.7, 2.835), ( 4.1, 4.305), (1.8, 1.890), (1.5, 1.575), (1, 1.050)

**Data 1:** Data 1 represents a COVID-19 data belonging to the Netherlands of 30 days, which have been recorded from 31 March to 30 April 2020. This data had been formed of rough mortality rate taken from Almongy et al.^17^. Advancements and perspectives in COVID-19 research are essential for addressing the multifaceted challenges posed by the pandemic, protecting public health, and building a more resilient and prepared society for the future. Recent studies have highlighted innovative computational diagnostics and severity analysis techniques for COVID-19, such as multilevel threshold image segmentation for chest radiography^18^, deep learning methods for diagnosing COVID-19 and its variant^19^, and analysis of COVID-19 severity using evolutionary machine learning approaches by Shi^20^. Su et al.^21^ provided a network based drug target set enrichment analysis method, which offered valuable insights into leveraging existing drugs for repurposing against COVID-19. Moreover, Xiong et al.^22^ investigated that the COVID-19 has accelerated the adoption of e-learning worldwide, with initiatives like ubiquitous e-teaching and e-learning gaining momentum.

**Data 2:** The second data set is taken from paper of Jamal et al. (2019). It represents therelief times of 20 patients receiving analgesic. The data values are given as follows in the Table 5.

**Figure 4:**
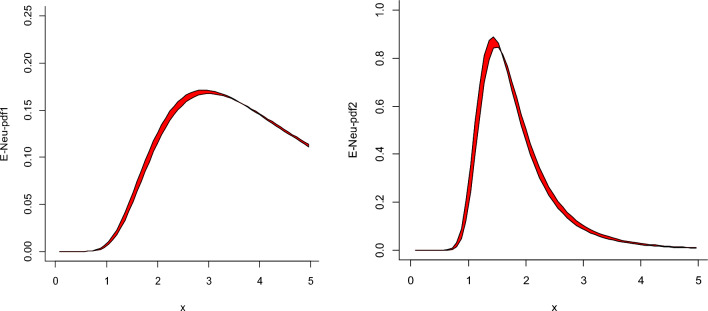
Estimated plots for the NeS-BrIIImodel for data sets 1 and 2, respectively.

**Figure 5:**
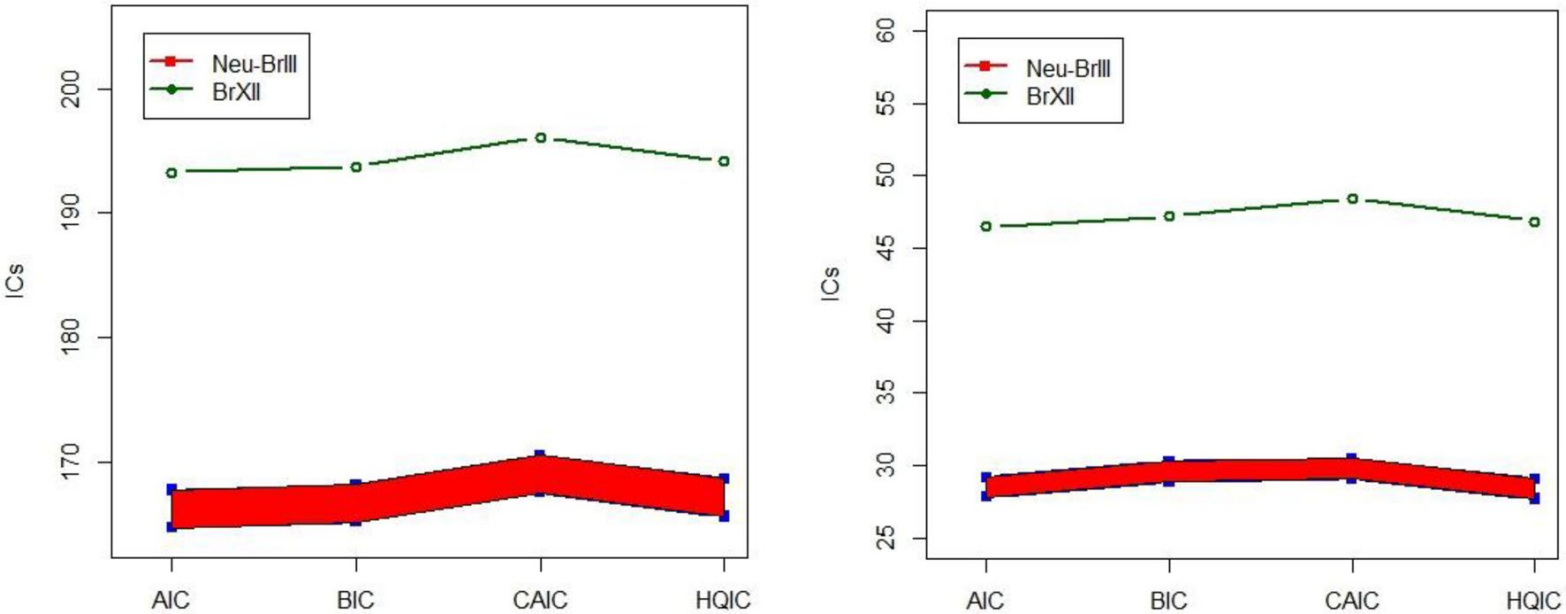
Plots of ICs for NeS-BrIII in contrast to BrXII model for data set 1 and data set 2, respectively.

Tables 6 and 7 revealed noteworthy observations: all information criteria associated with the NeS-BrIII model are consistently smaller when compared to the classical models. This comparison underscores the precision and reliability of the NeS-BrIII distribution’s results, as evident in the provided intervals. In stark contrast, the classical distributions exhibited unclear and ambiguous findings, primarily due to their inherent imprecision and errors. These limitations are notably absent in the NeS-BrIII distribution, making it a preferred choice for modeling and analysis. For a visual representation of the NeS-BrIII model’s performance, refer to Fig. 4, showcasing the fitted plots that further underscore its accuracy and suitability for practical applications. Further, we executed a visual comparison of NeS-BRIII model with classical BrXII model by plotting the numerical findings of these ICs in Fig. 5. The shaded region signifies the estimated interval values of the proposed NeS-BrIII model. Naturally, we can observe that the projected model yields least results in comparison to traditional BrXII model which is the prerequisite for all these ICs based on a trade-off between model intricacy and goodness of fit.

**Table 6:** MLEs and their standard errors (in parentheses) and goodness-of-fit statistics for models for data set 1.

Distribution	MLEs and standard errors (in parentheses)		Goodness-of-fit statistics for models			
	$\hat{\lambda }_{Neu}$	$\hat{\theta }_{Neu}$	AIC	CAIC	BIC	HQIC
NeS-BrIII I_Neu_ = [0, 0.05]	**[10.009, 10.636]** **(2.634, 2.862)**	**[1.655, 1.665]** **(0.198, 0.199)**	**[164.721,167.727]**	**[165.165,168.171]**	**[167.523,170.529]**	**[165.617,168.624]**
Burr-XII	7.51 (6.06)	0.080 (0.066)	193.26	193.71	196.07	194.16
W	0.026 (0.014)	1.86 (0.24)	164.70	165.52	167.87	165.97
NH	20.32 (22.03)	0.005 (0.006)	164.92	165.36	167.72	165.81

Significant values are given in bold.

**Table 7:** MLEs and standard errors (in parentheses) and goodness-of-fit statistics for models for data set 2.

Distribution	MLEs and standard errors (in parentheses)		Goodness-of-fit statistics for models			
	$\hat{\lambda }_{Neu}$	$\hat{\theta }_{Neu}$	AIC	CAIC	BIC	HQIC
NeS-BrIII I_Neu_ = [0, 0.05]	**[5.226, 6.043]** **(1.680, 2.044)**	**[3.897, 3.841]** **(0.736, 0.723)**	**[27.809,29.181]**	**[28.900,30.272]**	**[29.087,30.459]**	**[27.690,29.063]**
Burr-XII	37.47 (51.48)	0.045 (0.062)	46.47	47.18	48.46	46.86
W	0.121 (0.055)	2.785 (0.425)	45.17	45.87	47.16	45.561
NH	32.39 (99.36)	0.011 (0.036)	59.757	60.46	61.74	60.14

Significant values are given in bold.

## Conclusion

This study introduced a novel probabilistic model, the neutrosophic Burr-III distribution, tailored for applications in neutrosophic surface analysis. In the realm of real-world problem, our exploration of the neutrosophic Burr-III distribution has shown its aptitude in addressing data ambiguity and vagueness, an aspect where the classical Burr-III distribution falls short. The neutrosophic Burr-III distribution not only insights into reliability properties, moment expressions, order statistic, and entropy measures but also proves its versatility as a robust tool for deciphering complex data. To validate its practical significance, we applied the neutrosophic Burr-III distribution to real-world data sets, placing it in a head-to-head comparison with the well-known classical distributions. The outcomes of the analysis unequivocally demonstrated that the neutrosophic Burr-III distribution surpassed its classical counterpart in capturing the intricate nuances of the underlying data, signaling its potential as a superior modeling tool across various domains. Thus the neutrosophic Burr-III distribution opens up new avenues for addressing the complexities of real-world problems, where ambiguity is often the norm rather than the exception. This study also opens up several avenues for researchers and practitioners for future research in neutrosophic analysis and probabilistic modeling, ranging from the development of new models and algorithms paving the way for new insights, methodologies and applications in data analysis and decision making. However, this study acknowledges some limitations in specific contexts, such as theoretical and computational challenges. Moreover the study is valid only when the data is ambiguous and vague. If the data is crisp and well-defined the classical model is valid leaving no space for the neutrosophic analysis.

However, we acknowledge limitations in specific contexts, such as theoretical and computational challenges.

## Data Availability

The data used in the article is given therein.
